# ReMiDY (rehabilitation in mild stable degenerative cervical myelopathy): protocol for feasibility randomized controlled trial

**DOI:** 10.1038/s41393-025-01148-z

**Published:** 2026-02-10

**Authors:** Caroline Treanor, Ciaran Bolger, Ailish Malone

**Affiliations:** 1 Department of Physiotherapy, Beaumont Hospital, Dublin, Ireland; 2 Department of Neurosurgery, National Neurosciences Centre, Beaumont Hospital, Dublin, Ireland; 3 School of Physiotherapy, Royal College of Surgeons in Ireland, 123 St Stephens Green, Dublin, Ireland; 4 Department of Clinical Neurosciences, Royal College of Surgeons in Ireland, 123 St Stephens Green, Dublin, Ireland

**Keywords:** Rehabilitation, Randomized controlled trials

## Abstract

**Study design:**

A two parallel group exploratory single center feasibility randomised controlled trial. A nested qualitative study will explore the acceptability of the study processes and patient and physiotherapists experiences of the interventions.

**Objectives:**

The aim of this trial is to test the feasibility of undertaking an RCT investigating the effectiveness of a mult- component structured physical rehabilitation intervention aimed at reducing physical disability in adults with mild stable DCM compared with clinical surveillance.

**Setting:**

The study will take place in the national neurosurgical centre in Dublin, Ireland.

**Methods:**

A two parallel group exploratory single center feasibility RCT with will recruit 24 people with mild stable DCM from the outpatient clinics of a tertiary neurosurgical referral service. A nested qualitative study will explore the acceptability of the study processes and patient and physiotherapist experiences of the interventions. The intervention will include education, a physical activity behavioural change intervention, cervical range of motion exercises, neck, upper limb and scapular strengthening exercises and task specific hand function training. The primary outcomes are the feasibility of recruitment, adherence, and retention. The secondary outcomes which will be completed at baseline and 12 weeks follow up are the DCM core outcome set and an NRS for neck and arm pain.

**Conclusion:**

Developing novel rehabilitation interventions for people with DCM is the sixth most important research priority in the field of DCM. The findings from this feasibility RCT will inform the feasibility and design of a future definitive RCT.

**Sponsorship:**

Caroline Treanor is undertaking a professional doctorate for which she has received financial support from the Royal College of Surgeons in Ireland Strategic Academic Recruitment (StAR) programme.

## Introduction

Degenerative cervical myelopathy (DCM) is a standard unifying term for cervical myelopathy, the most common type of non-traumatic spinal cord injury, which occurs secondary to degenerative and or congenital changes to the structure of the spinal column [1]. The most up to date clinical practice guideline recommends surgical intervention for people with moderate or severe DCM, progressive DCM or those at high risk of progression [2]. The guideline also suggest that people with mild DCM should be offered surgery or a supervised trial of structured rehabilitation [2]. The recommendatons for people with mild DCM are based on low to very low quality evidence [3].

The decision to proceed with surgical intervention in mild DCM is conflicted due to the limited data available on the natural history of mild DCM [4], evidence that surgery is associated with at best small improvements in mild DCM [5–7] and the risk of a surgery related complicaton is high, irrespective of disease severity [6, 7]. To date, there have been no high quality studies investigating alternatives to surgery in DCM including rehabilitation [8]. An international multi-stakeholder research priority setting partnership has established that developing novel rehabilitation interventions for people with DCM both as a primary intervention or as an adjunct to surgery is the sixth most important research priority in the field of DCM [9].

People with mild DCM typically present with neck pain, neck stiffness, upper limb weakness and dexterity loss. There is evidence from other comparable clinical populations (i.e. stroke, traumatic spinal cord injury and cervical radiculopathy) that rehabilitation has the potential to deliver clinically important improvements in neck pain and related disability and upper limb function [10, 11]. Before an adequately powered RCT can be designed for mild DCM, the feasibility of participant recruitment and retention must be established, the interventions must be refined, the safety and outcome of the proposed physical rehabilitation intervention explored, and a sample size calculated [12]. Clinical surveillance is the current standard of care for people with mild DCM who have not reached the threshold for surgery or have elected to proceed with non-operative care. Therefore, clinical surveillance has been chosen as the comparator intervention in this trial. Therefore, the aim of this trial is to test the feasibility of undertaking a RCT investigating the effectivess of a physical rehabilitation intervention compared to clinical surveillance in adults with mild stable DCM.

## Objectives

The primary objectives are [1] to confirm the incidence of mild stable DCM in the neurosurgical outpatient clinics, [2] to assess the eligibility rate of people with mild stable DCM (defined as no worsening of neurological symptoms x 3 months), [3] to assess the participation rate of those eligible, [4] to establish participant and clinician adherence to the intervention and the acceptability of the interventions to participants and clinicians and [5] to determine participant retention, and the acceptability of the proposed outcome measures by measuring the proportion of patients providing complete outcome data at 12 weeks and the burden of measurement tool completion. The secondary objectives are [1] to describe changes and estimate variability in clinical outcomes to inform the sample size and design of a future effectiveness study and [2] to conduct a process evaluation according to Medical Research Council guidelines to assess fidelity and quality of implementation and clarify causal mechanisms [13].

### Trial design

**Fig. 1:**
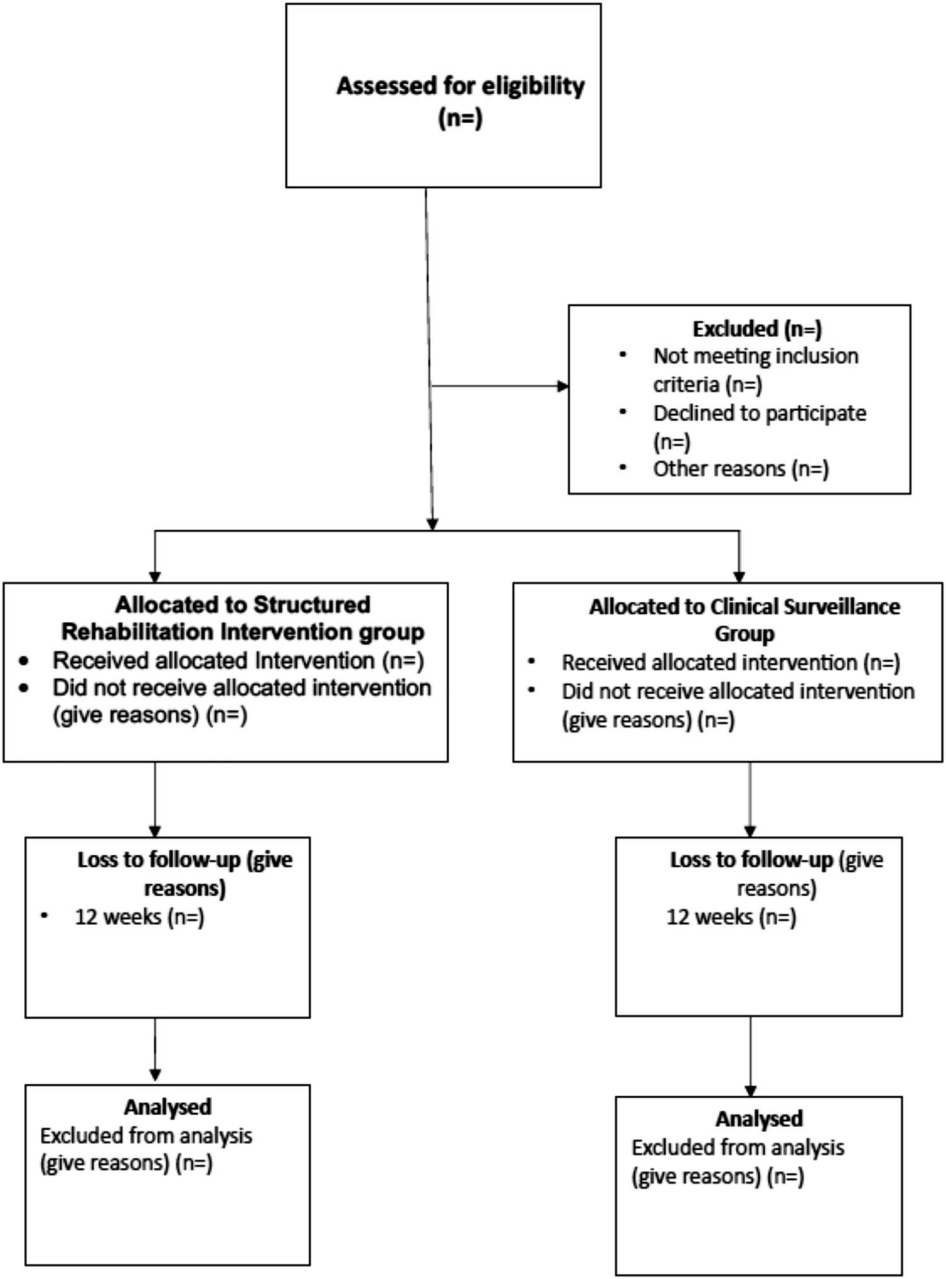
Sample participant flow diagram following CONSORT guidelines. This diagram shows the propsed flow of participants in the ReMiDY feasibility RCT and the data on recruitment, randomisation, retention and analysis that will be reported.

This trial is a two parallel group exploratory single center feasibility randomised controlled trial (Fig. 1). A nested qualitative study will explore the acceptability of the intervention to both participants and the treating physiotherapist using the theoretical framework of acceptability [13, 14]. The updated medical research council (UK) framework for developing and evaluating complex intervention was used in the development of this intervention[15]. The standard protocol Items: recommendations for interventional trials statement [16] and the template for intervention description and replication (TIDieR) checklist and guide [17] were used in the preparation of this protocol. This feasibility trial has been registered on clinicaltrial.gov (NCT06781658).

### Study setting

The study will take place in the Department of Neurosurgery in Beaumont Hospital which is large urban academic teaching hospital in Dublin, Ireland. Beaumont Hospital is the national referral service for neurosurgery and a regional spinal surgical service.

### Eligibility criteria

- **The inclusion criteria are:**
  - Participants aged ≥18 years.
  - Diagnosis of DCM (Clinical symptom of DCM + upper motor neuron sign + imaging evidence of cervical cord compression). Diagnosis will be confirmed by a consultant neurosurgeon [18].
  - Modified Japanese Orthopaedic assessment (mJOA) score of 15-17, indicating mild DCM. The mJOA is a clinician administered validated measure of DCM severity [19].
  - Adequate comprehension of English to understand the purpose of the study and give written informed consent
- **The exclusion criteria are:**
  - Patient or surgeon preference for urgent surgical decompression for progressive DCM, motor radiculopathy or severe unrelenting radicular arm pain or significant risk factors for progression including co-existing cervical radiculopathy and circumferential cord compression.
  - Diagnosis of other neurological condition that could confound assessment.
  - Involvement in another clinical study or trial.
  - Pregnancy.
  - If the participant is unable to commit to a 12-week programme of up to 10 sessions of supervised physical rehabilitation and participating in a regular home exercise programme for the duration of the study.

## Recruitment

Recruitment to the trial will be via the neurosurgical outpatient clinics in Beaumont Hospital. All new outpatient spinal referral letters to five consultant neurosurgeons who accept outpatient spinal referrals will be screened for any clinical or imaging evidence suggestive of DCM by the research team prior to the patients attending for their first assessment. The patient will then attend for their neurosurgical consultation where they will be screened for DCM and have a myelopathy severity index completed as part of their usual care.

If a patient is diagnosed with mild stable DCM at their neurosurgical clinic outpatient appointment and the surgical team and patient have made a shared decision to trial non operative care they will be advised about the study by their treating clinician and provided with a participant information leaflet. The patient’s verbal consent will be sought for a member of the research team to contact them by phone after a 48-h cooling-off period. A two-step screening process will be used to determine eligibility including a telephone screen, followed by a a face-to-face appointment with the researcher who is an experienced specialist physiotherapist in neurosurgery to facilitate the completion of their eligibility check, where they will have the opportunity to ask questions and, if willing to proceed, to sign informed consent. Baseline data will be collected at their first appointment with the study researcher. The baseline data will include all the relevant components of the DCM core data set and the ASIA/ISCoS international standards for neurological classification of spinal cord injury.

The revised physical activity readiness questionnaire will be used to identify any health-related risks of exercising that would require a participant to consult their general practitioner prior to participating in the physical rehabilitation intervention [20].

## Interventions

Participants in both groups will receive standardised verbal and written education on the natural history of DCM and indicators of neurological deterioration with clear instructions on how to contact their surgical team if they experience any neurological deterioration. They will be advised not to have any cervical spine manipulation or mobilisations undertaken due to the associated risk of spinal cord injury in people with established central canal stenosis [21]. They may seek other health care services, and they will be asked to record any contact with other healthcare services for their neck and upper limb symptoms on a logbook while they are enrolled in the trial.

## Clinical surveillance

Participants assigned to the clinical surveillance control group will be invited to attend a single, one to one education session which will be supplemented with a written patient information booklet after which they will be scheduled for a 12 week follow up.

## Structured Physical Rehabilitation

### Rationale and development of the intervention

The physical intervention is targeting the patient specific physical impairments and physical disability which occur secondary to DCM. Dynamic MRI, cadaveric, clinical, and biomechanical studies have been used to inform the design of the physical rehabilitation intervention as well as exercise prescription guidelines.

Participants will be prescribed a multi-component intervention which will include education, cervical range of motion exercises, progressive neck strengthening exercises, individualised scapular and upper limb strengthening, task specific hand function training and a physical activity behavioural change intervention. The TIDieR checklist has been used to provide a comprehensive description of this complex intervention [17]. The scientific rationale, prescription guidelines and dosage of each component of the intervention and a detailed description of the equipment, infrastructure, personnel and logistics involved in delivering this intervention are described in the supplementary material (Appendix C).

## Outcomes

Table 1 describes the primary and secondary outcomes and the mediators of change.

**Table 1:** Primary and secondary outcomes and mediators of change.

Primary	Secondary	Mediators of change
• Incidence of mild stable DCM in the neurosurgical OPD clinics. • The proportion of people with mild stable DCM who meet the eligibility criteria. • Recruitment rate (per month). • Participant and clinician adherence to the physical rehabilitation intervention. • Acceptability of the intervention to the participants and clinicians. • Burden of measurement tool completion. • Participant retention.	• Physical functioning (Physical component score of the Short Form 36). • Myelopathy severity (mJOA). • Neck pain related disability (Neck disability index). • Neck pain and arm pain severity (numerical rating scale). • Spinal adverse event (spinal adverse events severity scale).	• Cervical Range of motion • Manual dexterity (9 Hole peg test) • Grip strength • Neck Strength • Free living physical activity. • Timed up and go test

*DCM* degenerative cervical myelopathy, *OPD* outpatient department, *mJOA* modified japanese orthopedic association scoring system.

## Outcomes

Appendix A provides a detailed description of the measurement tools, specific measurement variables, analysis metrics, method of aggregation, time point for each measurement and explanation of clinical relevance of all the primary and secondary outcomes and the mediators of change. It also includes the COSMIN criteria for the secondary outcomes. The following methods will be used to standardise the collection of the mediators of change:

## Cervical Range of motion

Standardised testing procedures will be used to measure active CROM in flexion/ extension, side flexion and rotation using the CROM3 device (Performance Attainment Associates, USA). This study will use the testing procedure reported in a previous high quality study which demonstrated good intra-rater reliability when the measurements were conducted by an experienced clinician in people with neck pain [22]. These will include a standardised starting position, instructions to participants, number of practice repetitions, how the range is recorded and the use of verbal and manual cues by the assessor. The CROM has also demonstrated excellent criterion validity for measuring cervical ROM in the sagittal, frontal and horizontal plane when compared to a non-invasive optoelectronic motion measurement system [23].

## Cervical strength assessment

Cervical muscle strength will be assessed with hand held dynamometry “make tests” which are isometric hold tests where the participant cannot move the resisting force. This study will employ the testing procedure from a previous high quality study which demonstrated excellent intra rater reliability in normal subjects, as no study has investigated the intra rater reliability of hand held dynamometry in people with neck pain or in patients with cervical spine structural pathology [24, 25].

The participants will be tested in the supine position for flexion and side flexion and the prone position for extension. The test procedure will consist of three isometric contractions held for three seconds for cervical flexion, extension and right and left side flexion. A 30-second rest will be given between each contraction. The first of the three contractions for each motion will be submaximal (50% of maximal effort) to give the participant an opportunity to become acquainted with the motion. Two maximal contractions will then be performed, and the peak force of both maximal contractions will be recorded. Consistent procedures will be used for placing the dynamometer. The dynamometer will be centred on the forehead just superior to the eyebrows for flexion tests. For extension, it will be positioned slightly superior to the external occipital protuberance. To test right and left side bending, the pad or strap will be placed on the lateral aspect of the head just superior to the ear. The force values will be normalized to body weight (BW). The normalized force values from the two maximal effort tests will be averaged and used for analysis.

## Manual dexterity assessment

The nine hole peg test will be used to assess manual dexterity. It is the recommended manual dexterity measure in the National Institutes of Health (NIH) toolbox for the assessment of neurological and behavioral function. It has excellent intra-rater reliability when investigated in a large sample of normal people across the age span and in other neurological conditions [26, 27]. Its reliability has not been specifically assessed in people with DCM or spinal cord injury. Testing will follow a standardised protocol which will control the positioning of the test and the testing procedure. The score will be the total time in seconds to complete the task. Participants will complete a practice trial plus two-timed trials starting with their dominant hand. Total time in seconds to complete a trial will be recorded and the average of the two trials will be used.

## Grip strength measurement

Details of the dominant hand and the most affected limb will be recorded. The American society of hand therapists protocol for hand grip assessment will be used to standardise the assessment of hand grip strength [28]. Although the reliability of hand grip strength assessment has not been assessed in a DCM specific population, a systematic review and meta-analysis has reported excellent reliability in people with upper extremity and neurological conditions [29]. This testing protocol will specify the dynamometer, body position, instructions to participants, number of practice trials and number of trials performed [28]. Both maximum and mean grip strength will be reported.

## Free living physical activity

All participants will be asked to wear a wrist-worn triaxial accelerometer (Axivity AX3, Open Lab, Newcastle) for at least 4 days. The accelerometer will record how many minutes per day each participant is sedentary and engages in light, moderate and vigorous physical activities. This study will explore the change in average minutes of sedentary behaviour and physical activity before and after the intervention.

## Timed up and go test

The timed “Up & Go” will measure in seconds, the time taken by a participant to stand up from a standard armchair (approximate seat height of 46 cm), walk three meters, turn, walk back to the chair, and sit down again. The participant will wear their regular footwear. No physical assistance will be given. They will start with their backs against the chair with their arms resting on the chair’s arms. They will be in-structed that, on the word go,” they should get up and walk at a comfortable and safe pace to a line on the floor three meters away, turn, return to the chair, and sit down again. The participants will walk through the test once before being timed to become familiar with the test. The time taken to perform the test in seconds will be recorded [30]. A previous study has investigated the electromyographic characteristics, kinetics and kinematics of gait impairment in a cohort of people with moderate to severe cervical spondylotic myelopathy (CSM) compared to healthy controls ([31, 32]. They reported a specific altered motor strategy in people with CSM which indicated that paresis was a likely underlying contributor to their gait impairment. The results of these studies also suggested that balance impairment may contribute to the gait abnormalities seen in DCM. Therefore, this study will use the” Timed up and Go test” as a lower limb functional physical performance test which is influenced by lower limb strength, balance and walking speed. Although the psychometric properties of the “TUG” have not been investigated in DCM, previous studies have reported excellent intra rater and inter-rater reliability in ambulatory people with motor incomplete spinal cord injuries and other neurological populations [33]. The “TUG” is a valid measure of functional independence in ambulatory people with incomplete spinal cord injuries [34]. In addition, the “TUG “correlates more strongly with the walking index for spinal cord injury which is a validated measure of walking in spinal cord injury than a timed walk test [35].

## Participant Timeline

The baseline variables include all the relevant elements of the DCM core data and outcome set [36]. Participant will be followed up at 12 weeks post randomisation.

## Imaging variables

The recently published core outcome and data set for DCM clinical studies specifies several imaging parameters that should be reported in DCM clinical studies [36]. These include the use of MRI and CT imaging in the patient assessment, the level(s) of compression, pathology causing compression, amount of cord compression, presence of cord signal change, the presence or absence of a spinal cord syrinx, spondylolisthesis and radiological stability. They have also specified that adjacent segment degeneration and cervical spinal alignment should also be captured as core outcomes however these two variables are not relevant for patients managed non operatively [36]. The ReMiDy feasibility study will focus on MRI characteristics which can be assessed using valid and reliable methods as patients with mild stable DCM who are being managed non operatively do not always have dynamic radiographs undertaken as part of their routine neurosurgical care. Supine MRI does not provide a valid assessment of radiological stability, cervical spine alignment or spondylolisthesis and therefore these parameters will not be collected as part of this study.

This feasibility study will record the number of recruited patients who had a CT scan, MRI scan and dynamic radiographs undertaken as part of their neurosurgical assessment. The baseline imaging variables will be reported by a radiologist from MRI scans using the methodology described in Appendix B.

## Sample size

The investigators are aiming to recruit a total of 24 participants: 12 in the physical rehabilitation intervention group and 12 in the clinical surveillance group. Previous research has identified an incidence rate of approximately seven percent of people with mild DCM among new referrals for cervical degenerative pathology to the neurosurgical unit [37]. The justifications for the sample size include both feasibility and the gains in the precision about the mean for every unit increase in the sample size per group and the gain in precision around the variance for each gain in the degrees of freedom [38]. For the nested qualitative study, purposive sampling of both clinicians and a variety of participants will be used until data saturation [39].

## Sequence Generation

Simple randomisation will be used to generate the allocation sequence. It will be generated by an independent researcher using a computer-generated randomisation schedule.

## Allocation Concealment

The allocation will be concealed from the investigator who is conducting the enrolment and outcome assessments. The independent researcher who is responsible for the randomisation will not release the randomisation code to the treating physiotherapist electronically until the patient has been recruited into the trial.

## Implementation of sequence allocation

The blinded assessor will be a specialist physiotherapist in neurosurgery who will inform the independent researcher when a new participant has been enrolled, and their baseline measures collected. Once randomised, the independent researcher will provide the outcome of the randomisation to the treatment physiotherapist, who will inform the participant of their group allocation by phone. The treating physiotherapists will not be involved in the outcome assessment and will not inform the blinded assessor of the sequence allocation.

## Blinding

Every participant will have a unique identifier. Clinicians delivering the interventions and trial participants will not be blinded to group allocations due to the nature of the intervention. Participants will be asked in advance of attending for their 12 week follow up not to disclose their group allocation with the outcome assessor. Data analysts will be blinded to group allocation. Unblinding will only occur in exceptional circumstances when knowledge of the group allocation is essential for further management of the participant.

### Data collection methods

The process for data collection will be standardised and explicitly described in the trial manual. Training in recording data relevant to the assessment of intervention fidelity and adverse events will be included in the trial workshops for treating physiotherapists.

### Data management

A trial code book will be generated for raw, non-numeric data and a standard process will be developed and implemented to improve the accuracy of data entry and coding in the trial database which will be explicitly described in the trial handbook. The data will be periodically checked for errors and the source documentation will be used to verify the data accuracy as required.

### Statistical analysis

Statistical analysis will be performed using STATA (Version 19) software.

The data will be checked for normality, then appropriate descriptive analyses will be used to summarise participant characteristics and outcomes. Feasibility, the primary outcome of this study, will be evaluated by calculating the following recruitment, adherence, and retention metrics:

- Incident rate of mild DCM in new referrals to the neurosurgical outpatient clinic (reported per month of recruitment period).
- The proportion of people with mild DCM who were eligible for enrolment.
- The proportion of eligible people who consented to participate.
- The proportion of participants who attended their 12 week follow up in both intervention groups.
- The proportion of participants who attended their scheduled physiotherapy sessions in the physical rehabilitation group.
- The number of core intervention components included in each treatment session.

The quantitative process data will be analysed before trial outcomes to avoid bias interpretation[13]. Intervention effects will be represented by point estimates and their standard deviations. Confidence intervals will be calculated and will be interpreted with regards to the minimum clinically important difference. This data will be used to estimate a sample size required for a definitive trial, if appropriate [12].

Semi-structured interviews of the participants and clinicians will be audio recorded, transcribed verbatim and analysed thematically. The interviews will map to the theoretical framework of acceptability [14]. Findings will be reported according to the COREQ guidelines for qualitative research.

A traffic light system of green (go), amber (amend) and red (stop) which will specify the recruitment, adherence, retention, and signal of efficacy criteria for progression to a main trail will be specified a priori.

## Harms

A risk of a serious adverse event related to the physical rehabilitation intervention is minimal. All adverse events that occur after the participant has been enrolled in the trial until the 12 week follow up will be recorded. The treating physiotherapists will be prompted to enquire about any adverse events at every scheduled appointment with structured questions and recording the outcomes in a standardised data collection sheet. They will also undertake standardised assessments of participants neurological function at every scheduled session and will refer participants to their consulting neurosurgeon if there is any significant neurological deterioration. The investigators will determine the relatedness of an event to trial intervention based on a temporal relationship, as well as whether the event is unexpected or unexplained given the subject’s clinical course, the natural history of DCM and previous medical conditions. The investigators will suspend the trial if more than one serious adverse events of any kind occur, and these are related or caused by the physical rehabilitation intervention from the trial. If the cause of the events cannot be identified or addressed or is plausibly related to the intervention, the trial will be terminated.

### Ethical and Dissemination

This trial will be sponsored by the Royal College of Surgeons in Ireland. Ethical approval for this trial has been granted by the Beaumont Hospital Medical Research Ethics Committee (BHMREC) (Rec Ref 24/10). All participants will take part on a voluntary basis and will be informed that they can withdraw at any time without this influencing their subsequent treatment in the department of neurosurgery. The trial will comply with the latest Declaration of Helsinki.

The Trial Steering Committee will monitor and supervise the progress and safety of the trial towards its interim and overall objectives.

The research findings will be distributed to people with DCM and healthcare professionals though publication in academic journals, submission to national and international conferences, the media, professional groups and charitable organisations including myelopathy.org. The research team will work with people with DCM to ensure that the research summaries are accessible to the target audience. Following the collection and analysis of data on feasibility, acceptability and effectiveness, the intervention may require refinement in collaboration with all relevant stakeholders before moving to the next stage of evaluation.

## Supplementary information

Appendix A

Appendix B

Appendix C

Figure 2 (appendix C)

## Data Availability

No datasets have yet been generated or analyzed for this study protocol. Upon completion of the feasibility trial, anonymized data underlying the reported results will be made available from the corresponding author upon reasonable request, in accordance with institutional and ethical guidelines. Any data sharing will comply with participant consent and data protection regulations.
