# Supplementary material for: ReMiDY (rehabilitation in mild stable degenerative cervical myelopathy): protocol for feasibility randomized controlled trial
Source: Spinal Cord. 2026 Feb 10;64(3):296–302. doi: 10.1038/s41393-025-01148-z (PMC12975509; doi:10.1038/s41393-025-01148-z)
Supplement: Supplementary file 1 — Appendix A [file 41393_2025_1148_MOESM1_ESM.docx]

**Appendix A: Overview of primary, secondary and Mediators of change**

| Outcome type | Measurement tool | Specific Measurement variable | Analysis metric | Method of aggregation | Time point for each measurement | Explanation of clinical relevance |
| --- | --- | --- | --- | --- | --- | --- |
| Primary | Number of people diagnosed with mild stable DCM during the study period | Incidence of mild stable DCM attending national neurosurgical service | Number of people diagnosed with mild stable DCM/ number of NP seen | Proportion | Baseline | Feasibility of recruitment |
| Primary | Number of people with mild stable DCM who are willing to participate but do not meet the eligibility criteria. | The proportion of people with mild stable DCM who are willing to participate but do not meet the eligibility criteria | Number of people with mild stable DCM who are willing to participate but do not meet the eligibility criteria | Proportion | Baseline | Feasibility or recruitment |
| Primary | Participant recruitment rate | Number of participants recruited per month | Count | Count | Baseline | Feasibility of recruitment |
| Primary | Participant and clinician adherence to the rehabilitation intervention | Number of scheduled sessions attended by participants and intervention fidelity & number of core intervention components included in each treatment session | Number of treatment sessions attended/ number of treatment sessions specified in the protocol & number of core intervention components included in each treatment session. | Proportion | 12 weeks | Adherence to the intervention as prescribed in the protocol |
| Primary | Acceptability of the intervention to participants and clinicians | Semi- structured intervention and focus groups | Thematic analysis | Qualitative | 12 weeks | Acceptability of the intervention |
| Primary | Burden of measurement tool completion | Semi structured interview | Thematic analysis | Qualitative | 12 weeks | Burden of measurement tool completion |
| Primary | Retention | Loss to follow up rate and reasons for loss to follow up | Number of participants who completed their 12 week follow up/ total number of participants recruited and reasons for loss to follow up, | Proportion | 12 weeks | Loss to follow up rates |
| Secondary | Physical Component Score of the Short Form 36 | Physical functioning | Change Score | Mean / Median | Baseline and 12 weeks | DCM core outcome set |
| Secondary | mJOA | Myelopathy Severity Scale | Change score | Mean/ Median | Baseline and 12 weeks | DCM core outcome set |
| Secondary | Neck Disability Index | Neck pain related disability | Change score | Mean/ Median | Baseline and 12 weeks | DCM core outcome set |
| Secondary | Spinal adverse Events Severity Scale (Version 2) | Spinal adverse events grading scale which will be scored by treating physiotherapist | Number of participants who report adverse events (reported by grade) | Count | 12 weeks | DCM core outcome set |
| Mediator of change | Cervical range of motion | Range of cervical flexion/ extension and rotation | Change scores | Mean/ Median | Baseline and 12 weeks | Investigating the impact of cervical ROM exercise programme |
| Mediator of change | Manual dexterity | Nine-hole peg test | Change scores | Mean/ Median | Baseline and 12 weeks | Investigating the impact of task specific hand function training on dexterity |
| Mediator of change | Grip Strength | Dynamometer | Change scores | Mean/ Median | Baseline and 12 weeks | Investigate the impact of upper limb strength training on grip strength |
| Mediator of change | Neck strength | Hand held dynamometer | Change scores | Mean / median | Baseline and 12 weeks | Investigate the impact of progressive neck strengthening programme on neck muscle strength. |
| Mediator of change | Functional mobility | Timed up and go | Changes scores | Mean/ Median | Baseline and 12 weeks | Investigate the impact of physical activity prescription of functional mobility |
| Mediator of change | Free-living Physical activity | Accelerometer for 7 consecutive days from waking until returning to bed at night(25). | Accelerometry-based physical activity features (count-per-minute) will be derived | Mean/ Median | Baseline and 12 weeks | Investigate the impact of PA behavioural change intervention on free living physical activity. |

COSMIN criteria for the secondary outcome measures

| Tool | Outcome | Performed by | Validity | Reliability | Responsiveness | Interpretability |
| --- | --- | --- | --- | --- | --- | --- |
| SF-36 | Health related quality of life | Patient | Low | Unknown | High | -Yes |
| NDI | Neck pain related disability | Patient | High | Low | Low | Yes |
| NRS Arm | Arm pain severity | Patient | High | Unknown | Unknown | Yes |
| NRS Neck | Neck Pain Severity | Patient | High | Unknown | Unknown | Yes |
| mJOA | Myelopathy severity scale | Clinician | Low | High | High | Yes |
