# Supplementary material for: ReMiDY (rehabilitation in mild stable degenerative cervical myelopathy): protocol for feasibility randomized controlled trial
Source: Spinal Cord. 2026 Feb 10;64(3):296–302. doi: 10.1038/s41393-025-01148-z (PMC12975509; doi:10.1038/s41393-025-01148-z)
Supplement: Supplementary file 2 — Appendix B [file 41393_2025_1148_MOESM2_ESM.docx]

**Appendix B:** Methodology for radiology assessments.

| Imaging variable | Methodology | Reliability |
| --- | --- | --- |
| Level(s) of compression | - Cervical stenosis at each level from C3 to T1 will be evaluated on the T2-weighted sequence. The evaluation of the stenosis will be based on the classification of Muhle et al 1998. - Grade 0: Normal width of the spinal canal, no signs of anterior and posterior subarachnoid space narrowing. - Grade 1: partial obliteration of the anterior or posterior subarachnoid space or of both - Grade 2: complete obliteration of the anterior or posterior sub- arachnoid space or of both - Grade 3: Anterior or posterior cord impingement or both (pincer effect). | - Interrater reliability: k= 0.61, Interrater reliability: k=0.72 (Ko et al 2017) |
| Pathology causing compression | - The radiologist will report the predominant disease process causing excessing pressure on the spinal cord from the following list of pathological processes agreed by consensus to encompass DCM: - Cervical spondylosis, ossification of the posterior longitudinal ligament, ossification of the ligamentum flavum, Klippel Feil syndrome, diffuse idiopathic skeletal hyperostosis, degenerative disc disease and cervical stenosis) (Davies et al 2024). | - Reliability is not known |
| Amount of cord compression | - Maximum spinal cord compression (MSCC): - The diameter of the most compressed area of the spinal cord on midsagittal T2 WI MRI will be measured. - The diameter of two non-compressed areas above and below on the midsagittal T2 WI MRI will also be measured. - The formula proposed by Karpova et al (2013) will be used to calculate the MSCC (1 - diameter of compressed segment/ average diameter of the 2 non compressed segments) x 100. - Maximum spinal canal compromise (MCC). - The diameter of the region with the greatest reduction of spinal canal diameter on midsagittal T1WI MRI will be measured. - The diameter of a non-reduced reference area above and below on the midsagittal T1WI will also be measured. - The formula proposed by Karpova et al 2013 for maximum canal compromise will be used: (1- diameter of region with greatest reduction of spinal canal diameter/ average of two non-reduced diameter references) x100. | - The intra- and interobserver ICCs have been reported as 0.96 +/- 0.08 and 0.79 +/- 0.09 for T2 MSCC (Karpova et al 2013). - The intra and inter observer ICC’s were previously reported as 0.88 +/- 01 and 0.75 +/- 0.04 for the T1 MCC. (Karpova et al 2013). |
| Presence of cord signal change | - The radiologist will report on the absence or presence of intramedullary T2W hyperintensity change. - T1 signal abnormality will not be reported as the interrater reliability has only been shown to only be fair (K= 0.33; 95% CI, 0.04-0.62 (Karpova et al 2013). | - Interobserver reliability has been shown to be good (k =0.74; 95% CI, 0.62-0.86) for T2WI changes (Karpova et al 2013) - Interobserver reliability has been shown to be excellent (k=0.93; range 0.88-0.99) Fu et al 2016 |
| Syrinx | - The presence or absence of a spinal cord syrinx on mid-sagittal MRI. | - Reliability is not known |
