# Supplementary material for: ReMiDY (rehabilitation in mild stable degenerative cervical myelopathy): protocol for feasibility randomized controlled trial
Source: Spinal Cord. 2026 Feb 10;64(3):296–302. doi: 10.1038/s41393-025-01148-z (PMC12975509; doi:10.1038/s41393-025-01148-z)
Supplement: Supplementary file 3 — Appendix C [file 41393_2025_1148_MOESM3_ESM.docx]

**Rationale for developing physical rehabilitation intervention in DCM:**

This gap in evidence presents because to date, there have been no high quality studies investigating the effectiveness of rehabilitation in people with any grade of DCM severity, including mild DCM (1). Individuals with mild DCM are theoretically ideal candidates for rehabilitation interventions, as their spinal cord injury is incomplete. This results in significant neurological preservation below the level of the injury, offering substantial potential for recovery.

People with mild DCM have, by definition, symptoms or signs of cervical cord compression such as upper limb weakness and dexterity loss. In addition, they present with the sequalae of degenerative spinal column pathology including neck pain, cervical range restriction and local muscle atrophy (2, 3). There is evidence that rehabilitation has the potential to deliver clinically important improvements in neck pain and related disability and upper limb function in other comparable clinical populations (such as stroke, traumatic spinal cord injury and cervical radiculopathy) (4, 5). Unlike these comparable populations, however, the natural history of mild DCM is poorly understood. It is known that some people with mild DCM will experience neurological deterioration, but the proportion of people with mild DCM and the likelihood of individuals with mild DCM experiencing deterioration are currently not known(6).

Developing a novel rehabilitation intervention for people with DCM requires significant modifications to standard therapeutic approaches for mechanical neck pain and radiculopathy to avoid exacerbating the existing injury to the cervical cord that occurs with DCM and to adequately monitor participants for neurological deterioration related to either natural history or the intervention.

The proposed rehabilitation intervention, called ReMiDY, includes motor retraining as a central component. Motor retraining is a term used to describe exercise interventions which incorporate strength training and task specific training(7). There is evidence supporting its effectiveness in studies of animals with recent incomplete spinal cord injuries and in stroke survivors(8, 9). There is a large high quality multi-centre international clinical trial currently investigating the effectiveness of early and intensive motor training on neurological recovery in people with incomplete spinal cord injuries (8, 9, 10).

The location and severity of degenerative change and cord compression in addition to personal factors such as an individual’s central canal and cervical cord anatomy and kinematics, influence the nature, distribution and severity of physical impairments in DCM. The intervention description reflects the importance of individualising the intervention for each person with mild DCM in recognition of the variability in physical impairments and associated physical disability. A conceptual model of the ReMiDY intervention is presented in figure 2. Dynamic MRI, cadaveric, clinical, and biomechanical studies have been used to inform the design of the rehabilitation intervention as well as exercise prescription guidelines (11, 12).

Participants will be prescribed a multi-component structured rehabilitation intervention including (1) a physical activity behavioural change intervention, (2) cervical range of motion exercises, (3) progressive neck strengthening exercises, (4) individualised scapular and upper limb strengthening and (5) task specific hand function training. The TIDieR checklist has been used to provide a comprehensive description of this complex intervention (13). This paper will describe the rationale and evidence base behind each component of the intervention and then a detailed description of the materials, personnel, infrastructure and logistics required to deliver the intervention. The feasibility trial protocol is described in an accompanying paper.

1. **Rationale for the physical activity behavioural change intervention:**

Physical activity is crucially important for health and wellbeing. There is evidence that people with arthritic conditions are generally less active than controls (11). No studies have investigated the impact of DCM, mechanical neck pain or cervical radiculopathy on free living physical activity (PA). A previous study has investigated daily stepping activity in a related population, namely individuals with motor incomplete all cause spinal cord injuries classified by the American Spinal Injury Association (ASIA) Impairment Scale as C or D. The average daily step count was approximately 2,600 steps a day (14). Low levels of physical activity predispose individuals to an increased risk of cardiovascular and metabolic diseases such as obesity, dyslipidaemia and diabetes (15). Developing chronic health problems of this nature also negatively impacts life expectancy and quality of life in people with all cause spinal cord injuries (16).

A systematic review on the effectiveness of behaviour-change interventions to improve physical activity participation in individuals with a spinal cord injury recently reported that behaviour change interventions that provided practical support, were individualised and employed objective monitoring had a greater effect on physical activity levels than those that did not use these techniques (Watson et al 2023). Therefore, the behavioural change intervention in this trial will target the physical activity levels of participants who are not active and will incorporate these behavourial change techniques including individualising the physical activity prescription, the physiotherapist working with the participant to address barriers to physical activity participation and encouraging patients to objectively monitor their free-living physical activity levels.

1. **Rationale for the cervical range of motion exercises:**

DCM occurs secondary to degenerative cervical spinal column pathology. In cervical spine degeneration, the kinematic properties of the affected segments are altered, and mobility decreases in all planes of motion (17). Neck stiffness has been identified as one of the initial symptoms of DCM (18). A large prospective study has found a significant difference in the total range of motion in the sagittal plane in people with DCM when compared to asymptomatic age matched controls (2). A recent high quality systematic review with network metanalysis found that range of motion exercises showed uncertain or negligible effects for people with mechanical neck pain. The studies in this review excluded people with DCM. Significant cervical range of motion restriction is more prevalent in people with DCM than patients with simple mechanical neck pain and therefore it is hypothesized that range of motion exercise may proffer more benefit in terms of pain and disability reduction than that which is seen in a mechanical neck pain population.

Exercise prescription in DCM requires the rehabilitation clinician to recognise the impact of cervical spine motion on the dynamics of the cervical canal and the cervical cord, particularly in the setting of established cord compression. Dynamic MRI and cadaveric studies have demonstrated increased cervical cord compression in extension and reduced cord compression in flexion due to changes in the relative diameter of the canal and the cervical cord with motion (19, 20). Studies have also demonstrated that the lateral columns and anterior horns are deformed by mechanical stress in flexion when there is pathology in the anterior aspect of the canal (i.e. disc herniation, spondylotic bar) (21). The static compression evident on a supine MRI may also be aggravated by spinal malalignment or deformity which is more pronounced in the upright position and segmental hypermobility which can cause additional dynamic compression with motion(22).

The ROM exercises in the ReMiDY study have been designed to limit excessive or repetitive flexion and extension in the region of the mid-cervical spine where the stenosis secondary to DCM is most pronounced and most physiological flexion- extension motion occurs (17). This is typically from C4-C7. Therefore, cervical ROM exercises in this study will be limited to cervical rotation and cranio-cervical flexion and extension in supine as axial rotation is predominantly achieved at the C1–C2 segment (17). Participants will be counselled that they should limit their exercises to a range that does not provoke or aggravate their neurological or radiating symptoms. The dose of flexibility exercise will be in line with the European League against Rheumatism (EULAR) recommendations for people living with inflammatory arthritis and arthritis(12). Participants will be asked to repeat each flexibility exercise four times twice a day. They will be instructed to stretch to the point of feeling tightness or slight discomfort. Initially participants will be instructed to hold the stretch for 10 seconds with the hold time being increased incrementally up to 60 seconds providing there are no adverse effects. In view of the evidence that flexibility exercises are most effective when the muscle is warmed through either actively through light to moderate aerobic activity or passively through external methods such as moist heat, participants will be advised to exercise after showering or physical activity (11).

.

1. **Neck strengthening exercises:**

Several high quality RCT’s have demonstrated that neck strengthening exercises have a clinically important impact on neck pain severity and neck related disability in people with mechanical neck pain and radiculopathy (23); however, no studies have investigated the impact on neck strengthening exercises in people with DCM.

Previous imaging studies have reported that there is an association between the severity of cervical extensor fatty muscle infiltration and the severity of cord compression and canal compromise in DCM at the level of maximum canal compromise (24). Cervical lean muscle mass is positively associated with cervical muscle strength in patients with DCM (3). In view of the evidence of pathological change in the cervical spine muscle system in DCM, it is hypothesized that a progressive neck strengthening programme of adequate dosage would have a positive impact on neck pain and neck pain related disability levels in people with DCM.

The proposed progressive cervical spine strengthening programme will be undertaken with the head in midline and will not involve through range strengthening to limit repetitive motion at the site of maximum cord compression. Participants will be counselled that they should limit their exercise to an intensity that does not provoke or aggravate their neurological or radiating symptoms. Participants will be prescribed isometric resisted cervical flexion/ extension/ rotation and lateral flexion. They will initially be prescribed isometric resisted exercises in a supine position, but the intensity of the exercise and the position (sitting and then prone) will be progressed within their symptom tolerance.

There is no clear evidence of an optimal dosage of exercise for neck pain (25) as exercise dosage does not appear to predict changes in pain in disability in people with neck pain. Therefore, the dosage of exercise recommended for strengthening by EULAR in their physical activity guidelines for people with inflammatory arthritis and arthritis will be adopted (12). Participants will be prescribed three sets of 8-12 reps, three days a week with 1-2 minutes rests per set and performing the exercise at moderate velocity. A small increase (<10%) of load will be applied when the individual can perform the workload for 1-2 repetitions over the desired number. As muscle strength increase the number of repetitions will be increased to 10-25 repetitions to target endurance with 30 second to one minute rest periods between each set (26). The decision to focus on strength or endurance will be determined by the patients specific functional restrictions.

1. **Upper limb strengthening exercises:**

Atrophy of the anterior horns of the spinal cord associated with motor neuronal loss is one of the principle pathophysiological changes that occurs with DCM and the primary determinant of weakness in people with incomplete spinal cord injuries (27). However, there is also evidence of changes in muscle structure and function including fibre size, fibre phenotypes and fatiguability which also negatively affect the force generating capacity in people with incomplete spinal cord injuries (28).

The authors of the most up to date Cochrane review on exercise for mechanical neck pain which included twenty-seven trials (2485 analysed /3005 randomised participants), concluded that there is a role for strengthening exercises in the treatment of chronic neck pain, and cervical radiculopathy if these exercises are focused on the neck, shoulder and shoulder blade region(29). DCM and radicular pain or radiculopathy frequently occur concurrently, as the same degenerative pathology that causes central canal stenosis also impacts the patency of the exit foramen at the same level. Many scapular and shoulder muscles are innervated by the C5/6/7 nerve roots which are the spinal levels most frequently affected by DCM.

In addition to the lower motor neuron weakness associated with nerve root compression, a pyramidal pattern of upper limb weakness where the extensors are preferentially impacted is a known feature of cervical cord compression (30).

A typical neurological screening examination tests the strength of a single motor key point within each myotome however it can be clinically reasoned that rehabilitation professionals managing patients with DCM with or without nerve root compression should undertake a more comprehensive assessment of the scapular and upper limb muscles to establish the complete pattern and severity of motor weakness. For people with DCM this should include a comprehensive assessment of the intrinsic hand muscles as weakness in multiple intrinsic hand muscles (opponens pollicis, abductor digiti minimi and first dorsal interossei) has been found in people with mild DCM in addition to anterior deltoid weakness (31). This is to ensure that the full extent of any motor impairment is accurately identified and precisely targeted with strengthening exercises.

There is strong evidence of the effectiveness of strength training in people with spinal cord injuries with grade three or more strength, which is the severity of strength deficit typically seen in the mild DCM (32, 33). The strength training should be progressive involving high load and low repetitions. Patients will be prescribed a maximum of three strengthening exercises targeting the muscle groups with the most significant impairment to optimize adherence (34).

The principles of strength assessment in the international standards for the classification of spinal cord injuries motor examination guide will be used to standardise the clinicians muscle strength assessment(35). Each muscle will initially be tested for its ability to move through range against gravity. If the participant is unable to move through range against gravity, the test position will be altered to assess the muscle in a gravity eliminated position. If the patient has full range in an antigravity position, then the ability to push against external resistance will be assessed.

Each participants exercise programme will be individualised based on the strength assessment. Patients who are unable to perform full active range in an antigravity position, will be instructed in how to perform the exercise in a gravity assisted position (where technically feasible) and dosage of the exercises will be modified to low load and high repetitions. If participants can move through range against gravity, their exercises will be progressed through the addition of theraband or free weights. The dosage of the strengthening exercises will be line with the guidelines for strength training in untrained individuals from the American College of Sports Medicine (ACSM)(36). Previous research has demonstrated the effectiveness of the dosage recommended in the ACSM guidelines in people with incomplete spinal cord injuries(10, 33). Clinicians will be provided with a clear algorithm for exercise progression or modification (in the event of an increase in symptoms) to standardise the intervention.

1. **Task specific hand function training:**

Recovering arm and hand function is one of top recovery priorities of people with DCM (37). Hand function disturbance in the form of dexterity loss, clumsiness and grip strength loss are presenting symptoms of DCM seen early in the disease process (31). Previous research has demonstrated that people classified as mild on the mJOA in fact have moderate impairment of dexterity (31) however, there is tentative evidence that positive adaptation is possible. A single study has demonstrated that longer duration of symptoms predicted smaller self-perceived disability when controlling for upper limb impairment in mild DCM, with disability diminishing at nine months and greatly reduced at 12 months (38). This research finding, along with other emerging evidence of neuroplasticity following incomplete spinal cord injuries, suggest that clinical stability and adaptation can theoretically occur in DCM (39, 40).

Neuroplasticity is the nervous system’s capacity for structural and functional reorganisation. Activity dependent neuroplasticity through massed practice is a key therapeutic target in post-spinal cord injury rehabilitation programs (7). Mechanisms underlying adaptation and recovery of function in DCM have begun to be explored. Researchers employing transcranial magnetic stimulation and functional MRI identified that supraspinal and cortical changes may facilitate adaptation of neurological function in DCM (42,(40).

The motor retraining component of this intervention aims to accelerate and amplify adaptation by targeting the various reparative endogenous mechanisms that have been idenitified throughout the neuroaxis(41). These include alteration in cellular morphology and biochemical properties, the accumulation and proliferation of neural precursor cells and changes in the connectivity of neurons (41). Experiments conducted over the last number of decades in animals and humans have observed a role for activity in shaping and/ refining anatomical plasticity. They have also identified that functional recovery followiing rehabilitation training appears to be task specific and that extensive plasticity exists for long periods after an injury (42). To date, no studies have invesitgated the effectiveness of motor retraining in people with any severity of DCM.

It is important that rehabilitation is individualised to each person’s needs, The Patient Specific Function Scale will be used to identiy specific functional tasks which patients are having difficulty with which will be used as the basis for their assessment and intervention. The content of the proposed task specific hand function training intervention has been developed based on content of upper limb rehabilitation interventions from other neurological clinical populations which demonstrated large improvements on both impairment and activity limitation (5, 43, 44). These interventions incorporated several active ingredients, the most critical of which was the clinician’s analysis of movement and performance in activities of daily living. The focus of the rehabilitation clinician’s assessment is the identification of potential contributory impairments (such as muscle weakness, loss of joint range, sensory loss) which can be addressed with targeted exercise (lengthening and strengthening muscles to ensure they are at a biomechanical advantage to generate force and training sensory discrimination). The principles of motor learning will be applied which include movement practice as close to normal as possible, high number of repetitions, attention to the motor task and training specificity (45). Supervision and coaching are considered a key component of the intervention, therefore participants will be asked to attend for regular supervised exercise sessions.

The participants in this study will have mostly mild impairments and therefore the dosage of the upper limb rehabilitation programme will be titrated to reflect this. The optimal dose for rehabilitation in DCM is not known; however, previous studies of upper limb rehabilitation in stroke patients with moderate and severe upper limb impairment delivered therapy over 90-300 hours over 3-12 weeks duration. This intervention will be delivered over 12 weeks and our goal is to achieve 48 hours of therapy time over the duration of the intervention (this includes the time to undertake the other components of the intervention as they are complementary).

**Location of the intervention and intervention provider:**

The intervention will take place in the department of physiotherapy in Beaumont Hospital, which is a standard physiotherapy outpatient setting in a large teaching hospital. The equipment required will be limited to a plinth, theraband and dumbbells. Participants will be rehabilitated by senior or clinical specialist physiotherapist who have experience of working in the department of Neurosurgery in Beaumont Hospital. The clinicians involved are experienced in the assessment and rehabilitation of people with specific cervical spine pathology. All the physiotherapists participating as treating clinicians in the trial will undergo a standardised half day training programme.

**Intervention delivery and duration:**

Participants will attend for individual face to face physiotherapy sessions and will also be prescribed a home exercise programme which will take approximately 40 minutes a day, six days a week for 12 weeks to complete which is a cumulative target exercise dose of 48 hours of over the duration of the intervention. For the first two weeks of the intervention, participants will attend twice weekly for supervised exercise sessions in addition to undertaking a home exercise programme so that they achieve a cumulative exercise dose of 240 minutes per week. Participants will then attend for weekly supervised exercise sessions for the next three weeks and then once every two weeks for the last six sessions (total 10 sessions over 12 weeks). Participants will attend for frequent in-person supervised exercise therapy session to allow for close monitoring of their symptoms for the duration of the intervention. The first session will be a one-hour session, but all subsequent sessions will be 30 minutes (the therapist will be allocated 15 minutes after each exercise session to complete the study paperwork).

1. Tetreault LA, Rhee J, Prather H, Kwon BK, Wilson JR, Martin AR, et al. Change in Function, Pain, and Quality of Life Following Structured Nonoperative Treatment in Patients With Degenerative Cervical Myelopathy: A Systematic Review. Global Spine J. 2017;7(3 Suppl):42S-52S.

2. Machino M, Yukawa Y, Imagama S, Ito K, Katayama Y, Matsumoto T, et al. Age-related and degenerative changes in the osseous anatomy, alignment, and range of motion of the cervical Spine. Spine. 2016;41(6):476-82.

3. Fortin M, Wilk N, Dobrescu O, Martel P, Santaguida C, Weber MH. Relationship between cervical muscle morphology evaluated by MRI, cervical muscle strength and functional outcomes in patients with degenerative cervical myelopathy. Musculoskelet Sci Pract. 2018;38:1-7.

4. Mallard F, Wong JJ, Lemeunier N, Cote P. Effectiveness of Multimodal Rehabilitation Interventions for Management of Cervical Radiculopathy in Adults: An Updated Systematic Review from the Ontario Protocol for Traffic Injury Management (Optima) Collaboration. J Rehabil Med. 2022;54:jrm00318.

5. Ward NS, Brander F, Kelly K. Intensive upper limb neurorehabilitation in chronic stroke: outcomes from the Queen Square programme. J Neurol Neurosurg Psychiatry. 2019;90(5):498-506.

6. Nouri A, Tessitore E, Molliqaj G, Meling T, Schaller K, Nakashima H, et al. Degenerative Cervical Myelopathy: Development and Natural History [AO Spine RECODE-DCM Research Priority Number 2]. Global Spine J. 2022;12(1_suppl):39S-54S.

7. Ben M, Glinsky JV, Chu J, Spooren AI, Roberts S, Chen LW, et al. Early and intensive Motor Training for people with spinal cord injuries (the SCI-MT Trial): description of the intervention. Spinal Cord. 2023;61(11):600-7.

8. Martins A, Silva CM, Gouveia D, Cardoso A, Coelho T, Gamboa O, et al. Spinal Locomotion in Cats Following Spinal Cord Injury: A Prospective Study. Animals (Basel). 2021;11(7).

9. Lohse KR, Lang CE, Boyd LA. Is more better? Using metadata to explore dose-response relationships in stroke rehabilitation. Stroke. 2014;45(7):2053-8.

10. Harvey LA, Glinsky JV, Chu J, Herbert RD, Liu H, Jan S, et al. Early and intensive motor training to enhance neurological recovery in people with spinal cord injury: trial protocol. Spinal Cord. 2023;61(9):521-7.

11. Rausch Osthoff AK, Juhl CB, Knittle K, Dagfinrud H, Hurkmans E, Braun J, et al. Effects of exercise and physical activity promotion: meta-analysis informing the 2018 EULAR recommendations for physical activity in people with rheumatoid arthritis, spondyloarthritis and hip/knee osteoarthritis. RMD Open. 2018;4(2):e000713.

12. Rausch Osthoff AK, Niedermann K, Braun J, Adams J, Brodin N, Dagfinrud H, et al. 2018 EULAR recommendations for physical activity in people with inflammatory arthritis and osteoarthritis. Ann Rheum Dis. 2018;77(9):1251-60.

13. Hoffmann TC, Glasziou PP, Boutron I, Milne R, Perera R, Moher D, et al. Better reporting of interventions: template for intervention description and replication (TIDieR) checklist and guide. BMJ. 2014;348:g1687.

14. Saraf P, Rafferty MR, Moore JL, Kahn JH, Hendron K, Leech K, et al. Daily stepping in individuals with motor incomplete spinal cord injury. Phys Ther. 2010;90(2):224-35.

15. Paluch AE, Bajpai S, Ballin M, Bassett DR, Buford TW, Carnethon MR, et al. Prospective Association of Daily Steps With Cardiovascular Disease: A Harmonized Meta-Analysis. Circulation. 2023;147(2):122-31.

16. Sezer N, Akkus S, Ugurlu FG. Chronic complications of spinal cord injury. World J Orthop. 2015;6(1):24-33.

17. Lindenmann S, Tsagkaris C, Farshad M, Widmer J. Kinematics of the Cervical Spine Under Healthy and Degenerative Conditions: A Systematic Review. Ann Biomed Eng. 2022;50(12):1705-33.

18. Munro CF, Yurac R, Moritz ZC, Fehlings MG, Rodrigues-Pinto R, Milligan J, et al. Targeting earlier diagnosis: What symptoms come first in Degenerative Cervical Myelopathy? PLoS One. 2023;18(3):e0281856.

19. Lee Y, Kim SY, Kim K. A Dynamic Magnetic Resonance Imaging Study of Changes in Severity of Cervical Spinal Stenosis in Flexion and Extension. Ann Rehabil Med. 2018;42(4):584-90.

20. Breig A, Turnbull I, Hassler O. Effects of mechanical stresses on the spinal cord in cervical spondylosis. A study on fresh cadaver material. J Neurosurg. 1966;25(1):45-56.

21. Joaquim AF, Baum GR, Tan LA, Riew KD. Dynamic Cord Compression Causing Cervical Myelopathy. Neurospine. 2019;16(3):448-53.

22. Nouri A, Martin AR, Mikulis D, Fehlings MG. Magnetic resonance imaging assessment of degenerative cervical myelopathy: a review of structural changes and measurement techniques. Neurosurg Focus. 2016;40(6):E5.

23. de Zoete RM, Armfield NR, McAuley JH, Chen K, Sterling M. Comparative effectiveness of physical exercise interventions for chronic non-specific neck pain: a systematic review with network meta-analysis of 40 randomised controlled trials. Br J Sports Med. 2020.

24. Naghdi N, Elliott JM, Weber MH, Fehlings MG, Fortin M. Morphological Changes of Deep Extensor Neck Muscles in Relation to the Maximum Level of Cord Compression and Canal Compromise in Patients With Degenerative Cervical Myelopathy. Global Spine J. 2024;14(4):1184-92.

25. Wilhelm MP, Donaldson M, Griswold D, Learman KE, Garcia AN, Learman SM, et al. The Effects of Exercise Dosage on Neck-Related Pain and Disability: A Systematic Review With Meta-analysis. J Orthop Sports Phys Ther. 2020;50(11):607-21.

26. Bayles MP CS, Kellar GG,. American College of Sports Medicine issuing body, American College of Sports Medicine issuing body. ACSM’s exercise testing and prescription. . Second ed. Bayles MP CS, Kellar GG,, editor. Philadelphia, PA: Wolters Kluwer; 2023.

27. Nouri A, Tetreault L, Singh A, Karadimas SK, Fehlings MG. Degenerative Cervical Myelopathy: Epidemiology, Genetics, and Pathogenesis. Spine (Phila Pa 1976). 2015;40(12):E675-93.

28. Leech KA, Kim HE, Hornby TG. Strategies to augment volitional and reflex function may improve locomotor capacity following incomplete spinal cord injury. J Neurophysiol. 2018;119(3):894-903.

29. Gross A, Kay TM, Paquin JP, Blanchette S, Lalonde P, Christie T, et al. Exercises for mechanical neck disorders. Cochrane Database Syst Rev. 2015;1(1):CD004250.

30. Tetreault L, Kalsi-Ryan S, Benjamin D, Nanna-Lohkamp L, Garwood P, Martin AR, et al. Degenerative Cervical Myelopathy: A Practical Approach to Diagnosis. Global Spine J. 2022;12(8):1881-93.

31. Kalsi-Ryan S, Karadimas SK, Fehlings MG. Cervical spondylotic myelopathy: the clinical phenomenon and the current pathobiology of an increasingly prevalent and devastating disorder. Neuroscientist. 2013;19(4):409-21.

32. Harvey LA, Fornusek C, Bowden JL, Pontifex N, Glinsky J, Middleton JW, et al. Electrical stimulation plus progressive resistance training for leg strength in spinal cord injury: a randomized controlled trial. Spinal Cord. 2010;48(7):570-5.

33. Bye EA, Harvey LA, Gambhir A, Kataria C, Glinsky JV, Bowden JL, et al. Strength training for partially paralysed muscles in people with recent spinal cord injury: a within-participant randomised controlled trial. Spinal Cord. 2017;55(5):460-5.

34. Medina-Mirapeix F, Escolar-Reina P, Gascon-Canovas JJ, Montilla-Herrador J, Jimeno-Serrano FJ, Collins SM. Predictive factors of adherence to frequency and duration components in home exercise programs for neck and low back pain: an observational study. BMC Musculoskelet Disord. 2009;10:155.

35. International Standards for the classification of spinal cord injuries: Motor examination guide [Internet]. 2020.

36. Association ASI. International Standards for the classification of spinal cord injuries: Motor examination guide. 2020.

37. Davies B, Mowforth O, Sadler I, Aarabi B, Kwon B, Kurpad S, et al. Recovery priorities in degenerative cervical myelopathy: a cross-sectional survey of an international, online community of patients. BMJ Open. 2019;9(10):e031486.

38. Kalsi-Ryan S, Riehm LE, Tetreault L, Martin AR, Teoderascu F, Massicotte E, et al. Characteristics of Upper Limb Impairment Related to Degenerative Cervical Myelopathy: Development of a Sensitive Hand Assessment (Graded Redefined Assessment of Strength, Sensibility, and Prehension Version Myelopathy). Neurosurgery. 2020;86(3):E292-E9.

39. Zdunczyk A, Schwarzer V, Mikhailov M, Bagley B, Rosenstock T, Picht T, et al. The Corticospinal Reserve Capacity: Reorganization of Motor Area and Excitability As a Novel Pathophysiological Concept in Cervical Myelopathy. Neurosurgery. 2018;83(4):810-8.

40. Wang C, Laiwalla A, Salamon N, Ellingson BM, Holly LT. Compensatory brainstem functional and structural connectivity in patients with degenerative cervical myelopathy by probabilistic tractography and functional MRI. Brain Res. 2020;1749:147129.

41. Raineteau O. Plastic responses to spinal cord injury. Behav Brain Res. 2008;192(1):114-23.

42. Freund P, Curt A, Friston K, Thompson A. Tracking changes following spinal cord injury: insights from neuroimaging. Neuroscientist. 2013;19(2):116-28.

43. McCabe J, Monkiewicz M, Holcomb J, Pundik S, Daly JJ. Comparison of robotics, functional electrical stimulation, and motor learning methods for treatment of persistent upper extremity dysfunction after stroke: a randomized controlled trial. Arch Phys Med Rehabil. 2015;96(6):981-90.

44. Daly JJ, McCabe JP, Holcomb J, Monkiewicz M, Gansen J, Pundik S. Long-Dose Intensive Therapy Is Necessary for Strong, Clinically Significant, Upper Limb Functional Gains and Retained Gains in Severe/Moderate Chronic Stroke. Neurorehabil Neural Repair. 2019;33(7):523-37.

45. Krakauer JW, Kitago T, Goldsmith J, Ahmad O, Roy P, Stein J, et al. Comparing a Novel Neuroanimation Experience to Conventional Therapy for High-Dose Intensive Upper-Limb Training in Subacute Stroke: The SMARTS2 Randomized Trial. Neurorehabil Neural Repair. 2021;35(5):393-405.

Author Contributions: CT and AM conceived the work that led to the submission and drafted and revised the manuscript for important intellectual content. CB approved the final version.

Funding: Caroline Treanor is undertaking a professional doctorate for which she has received financial support from the Royal College of Surgeons in Ireland Strategic Academic Recruitment (StAR) programme.

Competing interests: CT is a member of the RECODE peri-operative rehabilitation research incubator. AM and CB have no other relevant competing interests.
