# Supplementary material for: ReMiDY (rehabilitation in mild stable degenerative cervical myelopathy): protocol for feasibility randomized controlled trial
Source: Spinal Cord. 2026 Feb 10;64(3):296–302. doi: 10.1038/s41393-025-01148-z (PMC12975509; doi:10.1038/s41393-025-01148-z)
Supplement: Supplementary file 4 — Figure 2 (appendix C) [file 41393_2025_1148_MOESM4_ESM.pdf]

Figure 1: A conceptual model of the structured rehabilitation intervention			
Participant Profiles	Individual Assessment	Intervention	Outcomes
• Cervical cord compression<br>• Clinical evidence of mild stable DCM.<br>• Neck pain<br>• Arm pain<br>• Cervical ROM restriction.<br>• Pathological changes in the cervical spine muscle system.<br>• Scapular muscle weakness<br>• Upper limb muscle weakness.<br>• Loss of hand dexterity<br>• Physical deconditioning<br>• Neck pain related disability<br>• Reduced quality of life.	Change processes:<br>• Identify DCM related physical impairments contributing to functional impairment.<br>• Supervised individualised exercised prescription to increase exercise confidence.<br><br>Active elements:<br>• Comprehensive neurological examination<br>• Identify direction of cervical ROM restriction<br>• Cervical muscle strength assessment.<br>• Scapular muscle strength assessment.<br>• Upper limb strength including grip strength assessment<br>• Hand dexterity assessment<br>• Free living physical activity assessment<br>• Assist the patient in completing the patient specific functional scale to identify specific functional tasks that the patient is having difficulty with.<br>• Analysis of movement in functional tasks.	Change process:<br>• Increase free living physical activity.<br>• Increased cervical ROM<br>• Increased cervical muscle strength<br>• Increased scapular/ upper limb muscle strength<br>• Improved dexterity<br>• Increase exercise confidence<br>• Improve patients understanding of DCM, the natural history of DCM and warning signs of neurological deterioration.<br><br>Active elements<br>• Education<br>• Modified cervical ROM exercises<br>• Modified Neck strengthening exercises<br>• Scapular strengthening exercises<br>• Upper limb strengthening exercises<br>• Hand function task specific training<br>• Physical activity prescription with behavioural change intervention<br>• Feedback and reassurance	• Reduced neck and or arm pain<br>• Increase free living physical activity<br>• Improved understanding of DCM, natural history and warning signs of neurological deterioration.<br>• Reduced neck and arm pain related disability<br>• Improved quality of life<br>• Decreased health care utilization.
